# Effects of Jianpi therapy for cancer-related fatigue:a meta-analysis of randomized controlled trials

**DOI:** 10.3389/fonc.2025.1512460

**Published:** 2025-01-24

**Authors:** Jiaxing Dai, Huili Shui, Yuan Wu, Huanghui Zhang, Yuanyin Li, Shaowang Zhang, Bing Yang, Dongxin Tang

**Affiliations:** ^1^ The First Clinical Medical School of Guizhou University of Chinese Medicine, Guiyang, China; ^2^ Talent Base for TCM Tumor Inheritance and Science and Technology Innovation of Guizhou Province (Guizhou University of Chinese Medicine), Guiyang, China; ^3^ The First Affiliated Hospital of Guizhou University of Traditional Chinese Medicine (Guizhou Provincial Hospital of Chinese Medicine), Guiyang, China; ^4^ The Second Affiliated Hospital (Guangdong Provincial Hospital of Chinese Medicine), Guangzhou University of Chinese Medicine, Guangzhou, China

**Keywords:** Jianpi, traditional Chinese medicine, cancer-related fatigue, randomized controlled trials, meta-analysis

## Abstract

**Purpose:**

The Jianpi therapeutic strategy in traditional Chinese medicine aims to enhance the spleen’s digestive function and overall wellness. It has shown promise in improving cancer-related fatigue (CRF). This research systematically evaluates the effectiveness of Jianpi therapy in reducing fatigue in cancer patients through a meta-analytic review.

**Methods:**

An exhaustive search was performed within PubMed, Embase, Web of Science, Cochrane Library, SinoMed, Wanfang Data, China Science and Technology Journal Database, and China National Knowledge Infrastructure (CNKI) for randomized controlled trials concerning the application of Jianpi therapy to address CRF. The search spanned from the commencement of each database’s records to April 1, 2024. The extracted data were subjected to analysis using Stata (Version 15.1), with the selection of either a random-effects or fixed-effects model based on the heterogeneity among studies. Outcome measures were demonstrated with standardized mean differences (SMDs) or mean differences (MDs), and each complemented by a 95% confidence interval (CI). The Cochrane Risk of Bias Assessment Tool 2.0 was utilized to assess the potential biases within the studies.

**Results:**

A comprehensive analysis was performed on 45 eligible studies, all of which were conducted within China and encompassed a total of 3,596 participants. The meta-analysis indicated that Jianpi decoction alone exhibited the most significant improvement in the proportion of CD4 cells (SMD=1.34, 95% CI 0.54 to 2.31, P<0.001) and hemoglobin (MD=7.45, 95% CI 4.18 to 10.72, Z=4.47, P<0.001), while also more significantly reducing Piper Fatigue Scale scores (SMD=-2.05, 95% CI -2.71 to -1.39, P<0.001). The combined therapy, which integrated Jianpi therapy with standard care, demonstrated the greatest advantage in enhancing the proportion of CD3 cells (SMD=1.25, 95% CI 0.46 to 2.04, P<0.001). Furthermore, Jianpi therapy was found to be effective in lowering tumor necrosis factor-alpha levels (MD=-7.79, 95% CI -11.24 to -4.34, P<0.001) and concurrently enhancing interferon-gamma (MD=5.15, 95% CI 3.20 to 7.09, P=0.002), interleukin-2 (MD=8.37, 95% CI 6.14 to 10.59, P<0.001).

**Conclusion:**

Our research indicates that Jianpi therapy effectively alleviates CRF, reduces inflammation, and strengthens immune function. However, further high-quality, multicenter randomized controlled trials are essential to confirm these findings and strengthen the evidence.

**Systematic review registration:**

https://www.crd.york.ac.uk/PROSPERO/, identifier CRD42024566739.

## 1 Introduction

Cancer poses a substantial global health burden, with an estimated 20 million new diagnoses and nearly 10 million cancer-related fatalities annually (1). In the spectrum of issues linked to cancer and its treatment protocols, cancer-related fatigue (CRF) stands out as a widespread and disconcerting side effect (2). Recent epidemiological studies indicate that fatigue occurrence among cancer patients undergoing treatment varies widely, with estimates ranging from 25% to as high as 99%. This variability is dependent on factors such as the specific patient group, the therapeutic approach employed, and the methods used for fatigue assessment (2, 3). The National Comprehensive Cancer Network (NCCN) delineates CRF as a persistent and distressing subjective experience of physical, emotional, and cognitive fatigue that is disproportionate to recent activity, adversely affecting daily functioning (4). Unlike common fatigue, CRF significantly diminishes an individual’s quality of life, leading to reduced physical engagement, psychological strain, and impaired social relationships. The etiology and pathogenesis of CRF are complex and not fully understood, thought to stem from a combination of factors including cytokine secretion, systemic inflammation, immune system imbalance, disruptions in energy metabolism, and neuroendocrine changes (5–8). Additionally, the negative consequences of cancer therapies, including chemotherapy, radiotherapy, and targeted treatments, intensified the emergence and duration of CRF, thereby magnifying the difficulties encountered by individuals with cancer (9, 10).

In the current clinical landscape, the primary therapeutic modalities CRF encompass psychoeducational interventions, pharmacological therapies, and adjunctive treatment strategies (11–13). However, these approaches are constrained by several limitations, including the placebo effect, variability in individual patient responses, and the small sample sizes characteristic of many studies, which may limit the generalizability of these treatments to all patients experiencing CRF (14). In Traditional Chinese Medicine (TCM), the therapeutic approach of “fortifying” or “rejuvenating the spleen” is designed to bolster the organ’s capabilities, which are deemed essential for the metabolism and distribution of nutrients derived from food (15). Cancer patients frequently encounter CRF, a multifaceted symptom that persists despite rest and can markedly diminish their quality of life. TCM addresses CRF by targeting the underlying deficiencies and imbalances that lead to this exhaustion (16). The strategy of “fortifying the spleen” is commonly integrated into a holistic treatment plan that may encompass herbal remedies, dietary adjustments, acupuncture, and additional TCM practices (17). A thorough systematic review, which included 82 randomized controlled trials (RCTs), demonstrated that TCM interventions have a positive effect on mitigating CRF, particularly when compared with control groups (18).

TCM has been utilized to alleviate CRF, with the concept of ‘strengthening the spleen’ (Jianpi) emerging as a pivotal strategy in treatment. Nevertheless, the classification of Jianpi techniques and a comprehensive assessment of their therapeutic outcomes warrant revision, sparking discussions on the clinical effectiveness of TCM’s Jianpi interventions for CRF (19, 20). To bridge these knowledge gaps, we initiated a meta-analysis of RCTs to evaluate the efficacy of TCM’s Jianpi therapy in improving CRF. This systematic review aimed to offer a more sophisticated insight into the possible therapeutic advantages of TCM’s Jianpi approach within the scope of CRF treatment, shedding light on its prospective contribution to oncological care.

## 2 Materials and methods

### 2.1 Protocol registration

Our research protocol was duly registered with the International Prospective Register of Systematic Reviews under the identifier CRD42024566739.

### 2.2 Data sources and search methodology

We conducted a comprehensive search across PubMed, Embase, Web of Science, Cochrane Library, SinoMed, Wanfang Data, China Science and Technology Journal Database, and China National Knowledge Infrastructure from their inception up to April 1, 2024. Our search strategy aimed to identify all relevant studies assessing Jianpi therapy for CRF, encompassing terms such as cancer-related fatigue, tumor-related fatigue, chronic fatigue syndrome, fatigue, CRF, asthenia, tiredness, weariness, exhaustion, and various herbal treatments. As for Jianpi therapy, the search strategy included terms such as ginseng (Panax), licorice root (Glycyrrhiza uralensis), Astragalus (Huang Qi), and other traditional Chinese herbs, along with phrases like ‘strengthening the spleen’, ‘replenishing the spleen’, and ‘invigorating the spleen’. The complete search strategy is detailed in **Supplementary Table 1**. We imposed no language restrictions. Additionally, we scrutinized pertinent review articles and their citations.

### 2.3 Study selection process

We utilized Endnote X9 software to manage and de-duplicate the eligible studies identified during the literature screening process. Studies were included if they met the following criteria (1): Involving adults aged 18 years or older diagnosed with any stage of cancer, with no restrictions on sex, ethnicity, or healthcare setting (2); Incorporating Jianpi therapy as part of the intervention (3); Being RCTs. Exclusion criteria included (1): Mon-empirical literature such as reviews, correspondence, re-analyses, or conference abstracts (2); Lack of accessible source data or inability to calculate the mean difference (MD), or standardized mean difference (SMD) from the provided data. Two researchers independently assessed the studies against these criteria, with disagreements resolved through discussion or arbitration by a third party.

### 2.4 Data extraction and quality assessment

We strictly followed the Preferred Reporting Items for Systematic Reviews and Meta-Analyses (PRISMA) guidelines, as outlined in **Supplementary Tables 2**, **3** (21). Two researchers independently extracted data from the selected studies using a standardized Excel 2019 template, ensuring consistency. Extracted data included authorship, publication dates, patient demographics, treatment duration, and outcome measures. Discrepancies were resolved by a third reviewer. The potential for bias was assessed using the Cochrane Risk of Bias 2.0 tool (22), considering factors such as sequence generation, allocation concealment, participant and personnel blinding, missing data, reporting bias, and other sources of bias. The robustness of the evidence was evaluated by two experienced researchers using the GRADE-profiler software (version 3.6, The GRADE Working Group, 2010), taking into account the risk of bias, heterogeneity, indirectness, precision, and publication bias. Evidence quality was categorized into high, moderate, low, and very low (23). Continuous data were analyzed using MD, SMD, and 95% CI. For continuous data with different units, we used SMD for the analysis. Heterogeneity was assessed using the chi-squared test and the I² statistic, with an I² value above 50% indicating significant heterogeneity. In cases of substantial heterogeneity, a random-effects model was applied, and subgroup analyses were conducted to investigate potential sources. Publication bias was assessed using funnel plots and Egger’s test for studies with 10 or more trials (24, 25), with the trim-and-fill method employed to quantify its impact (26).

Sensitivity analyses were performed by sequentially excluding individual studies to evaluate the robustness of the overall effect estimates. All statistical analyses were conducted using Stata (version 15.1), with a significance threshold of P < 0.05 for two-tailed tests. Furthermore, trial sequential analysis (TSA) was conducted using TSA software version 0.9 to evaluate the impact of Jianpi therapy on fatigue scores, with a type I error rate of 0.05 and a type II error rate of 0.1, to validate the findings and mitigate the risk of false positives due to random chance (27).

## 3 Results

### 3.1 Search outcomes

**Figure 1:**
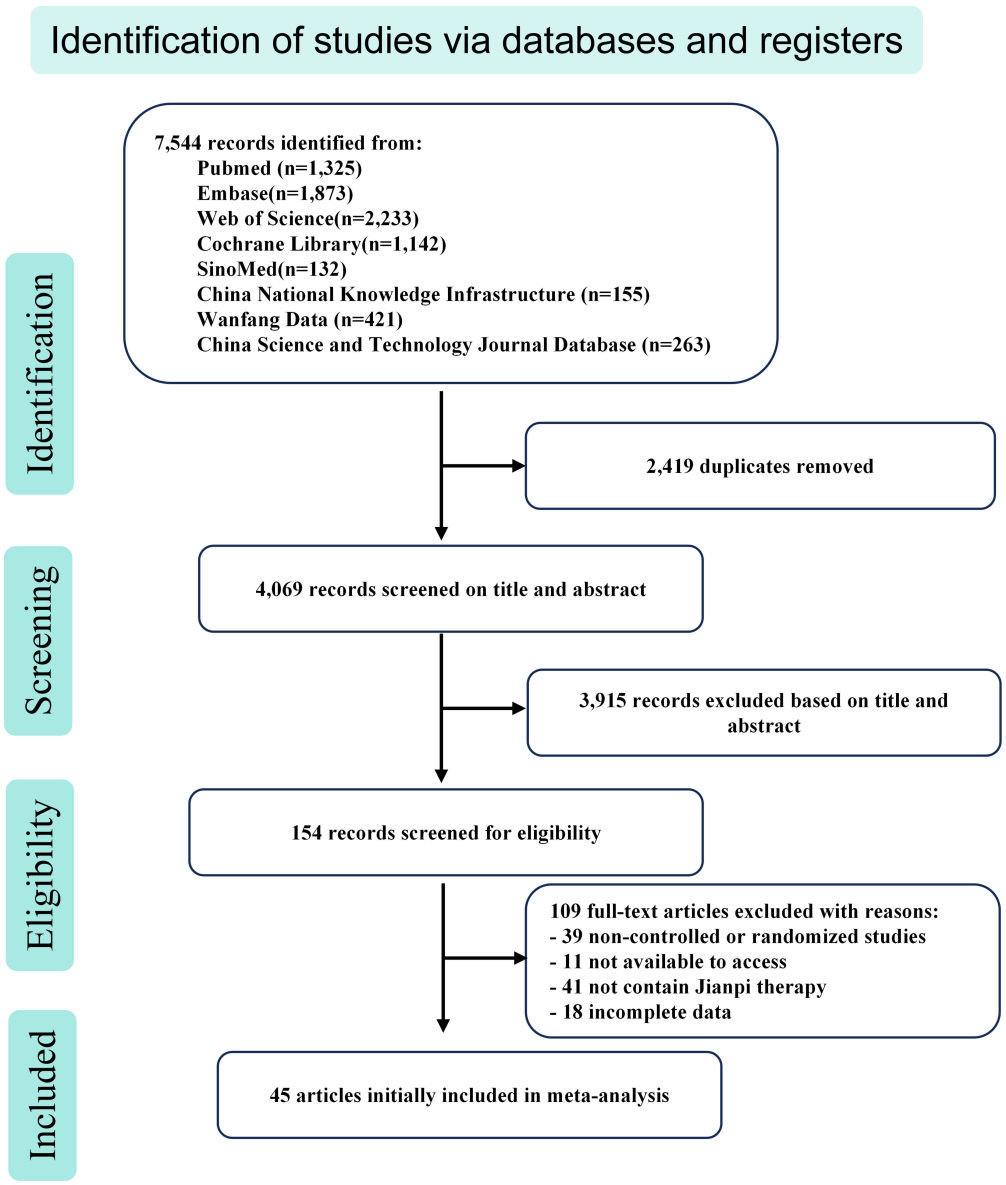
Flow chart of study identification, inclusion, and exclusion.

Our initial search yielded a total of 7,544 articles that appeared to be relevant. After removing 2,419 duplicate records, we were left with 4,069 papers for further evaluation. Upon reviewing the titles and abstracts, 154 full-text papers were retrieved for detailed assessment. Subsequently, 109 publications were excluded for the following reasons: non-controlled or non-randomized studies (n = 39), inaccessible articles (n = 11), absence of Jianpi therapy (n = 41), and incomplete data (n = 18). Ultimately, the meta-analysis encompassed 45 eligible articles, as depicted in **Figure 1**.

### 3.2 Overview of included studies

A total of 45 studies met the inclusion criteria, all of which were conducted in China and collectively enrolled 3,596 participants. Among these, one study featured a randomized trial comparing a placebo against a spleen-strengthening intervention (28). The 29 studies employed the traditional Chinese medicine Jianpi decoction as the intervention in the experimental group, while the control group received Western medical treatments, encompassing chemotherapy or primary care (29–57). In six studies, a Jianpi preparation was utilized within their experimental protocols (58–63). Additionally, two studies incorporated traditional Chinese medicine Jianpi plasters into their treatment regimens (64, 65). Ultimately, a diverse array of therapeutic approaches, encompassing the spleen-fortifying technique from traditional Chinese medicine, was employed in seven investigations to alleviate fatigue in cancer patients (66–72). **Supplementary Table 4** delineates the fundamental features of the encompassed research studies.

### 3.3 Meta-analytic review

#### 3.3.1 Impact of spleen-strengthening therapy on the piper fatigue scale

**Figure 2:**
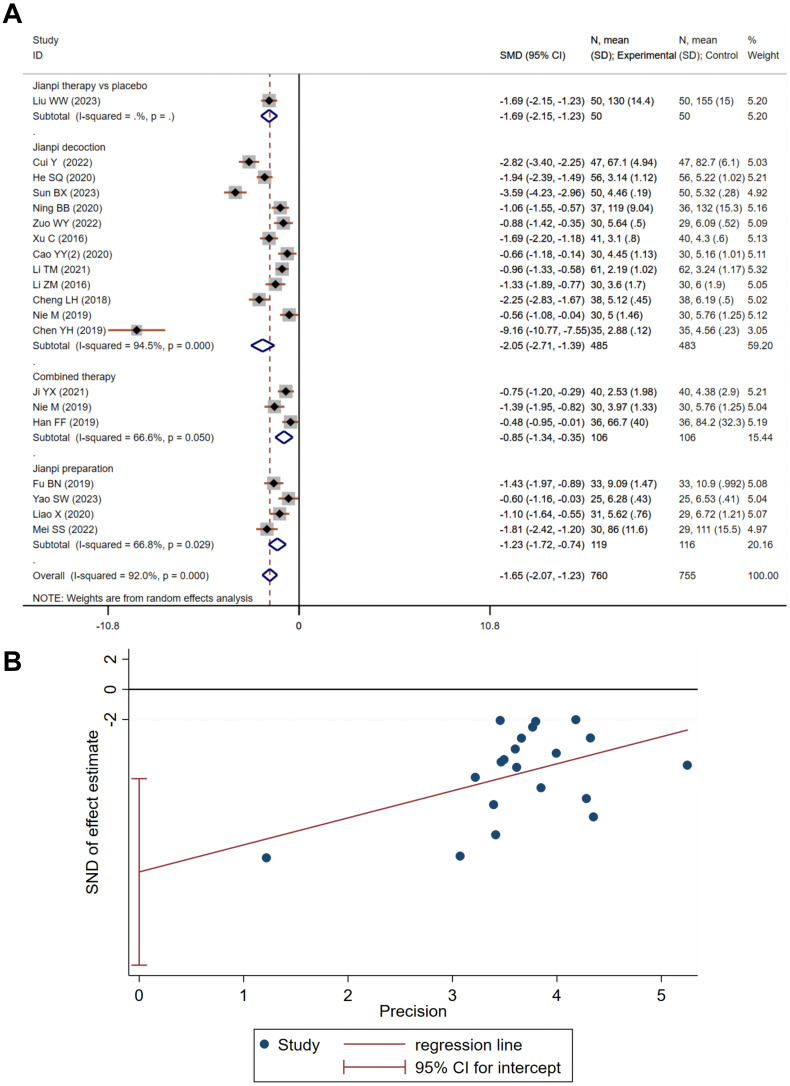
The effect of Jianpi therapy on PFS. **(A)** Forrest plot. **(B)** Egger’s test.

The PFS, a visual analogue self-assessment tool, indicates higher scores are associated with increased CRF severity. A meta-analysis of 20 studies, encompassing 1,515 subjects—755 in the treatment group and 760 in the control—revealed considerable variability (Heterogeneity test: Chi^2^ = 238.12, P<0.001, I^2^ = 92.0%). Utilizing a random-effects model, the aggregated SMD was -1.65 (95% CI -2.07 to -1.23), signifying a robust treatment effect (Z=8.02, P<0.001). Subgroup analysis favoured Jianpi decoction, yielding a more pronounced improvement in PFS scores (SMD=-2.05, 95% CI -2.71 to -1.39, Z=6.08, P<0.001, I^2^ = 94.5%). This suggests a significant therapeutic advantage over the control intervention (**Figure 2A**). Despite publication bias detected by Egger’s test (P<0.001, **Figure 2B**), the trim-and-fill method confirmed the robustness of the findings, adjusting the estimate to -2.097 (95% CI -2.560 to -1.635) after imputing data for six missing studies.

#### 3.3.2 Impact of spleen-strengthening therapy on anemia

**Figure 3:**
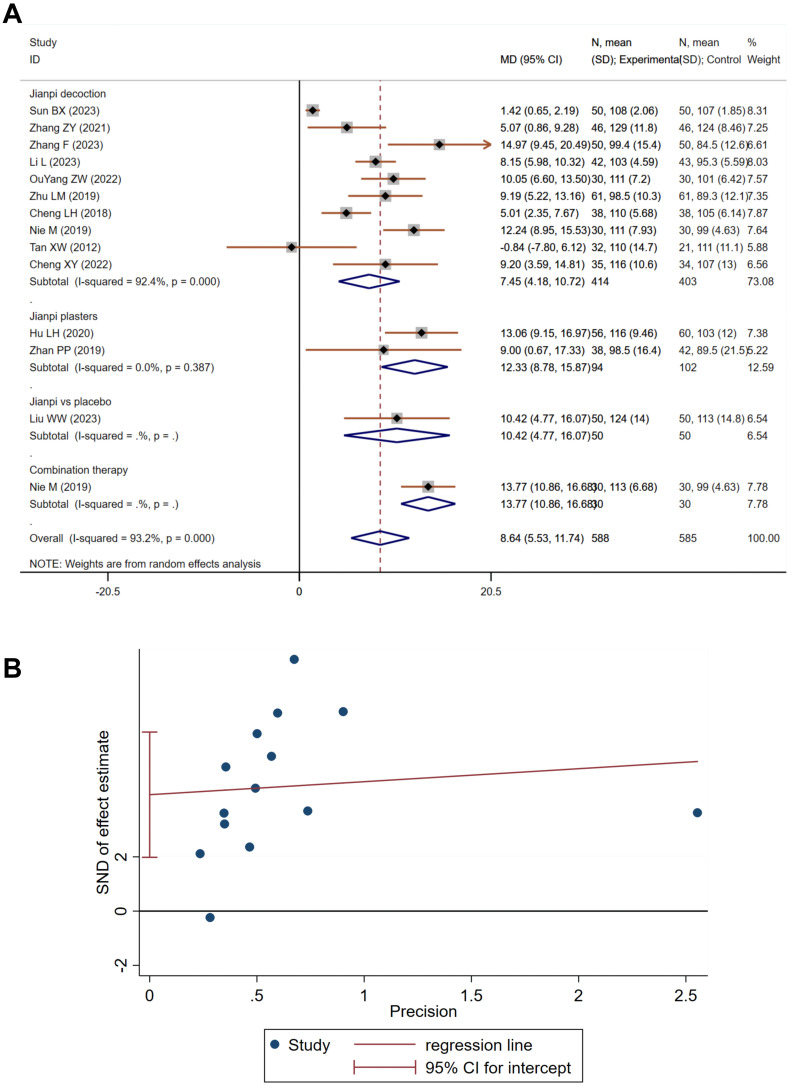
The effect of Jianpi therapy on anemia in CRF. **(A)** Forrest plot. **(B)** Egger’s test.

Anemia, defined by reduced hemoglobin levels, is prevalent in cancer patients and exacerbates fatigue and diminishes quality of life. A synthesis of 14 studies, involving 1,173 individuals—588 in the treatment and 585 in the control—demonstrated substantial heterogeneity (Heterogeneity test: Chi^2^ = 190.54, P<0.001, I^2^ = 93.2%). The random-effects model aggregated MD was 8.64 (95% CI 5.53 to 11.74), indicating a significant hemoglobin increase in the treatment group (Z=5.45, P<0.001, **Figure 3A**). Subgroup analysis by formulation reduced heterogeneity, implicating formulation diversity as a source of variability. Publication bias was evident on Egger’s test (P=0.002, **Figure 3B**), yet the trim-and-fill method, after accounting for two imputed studies, upheld the original estimate at 7.679 (95% CI 4.909 to 10.449).

#### 3.3.3 Impact of spleen-strengthening therapy on cytokines

**Figure 4:**
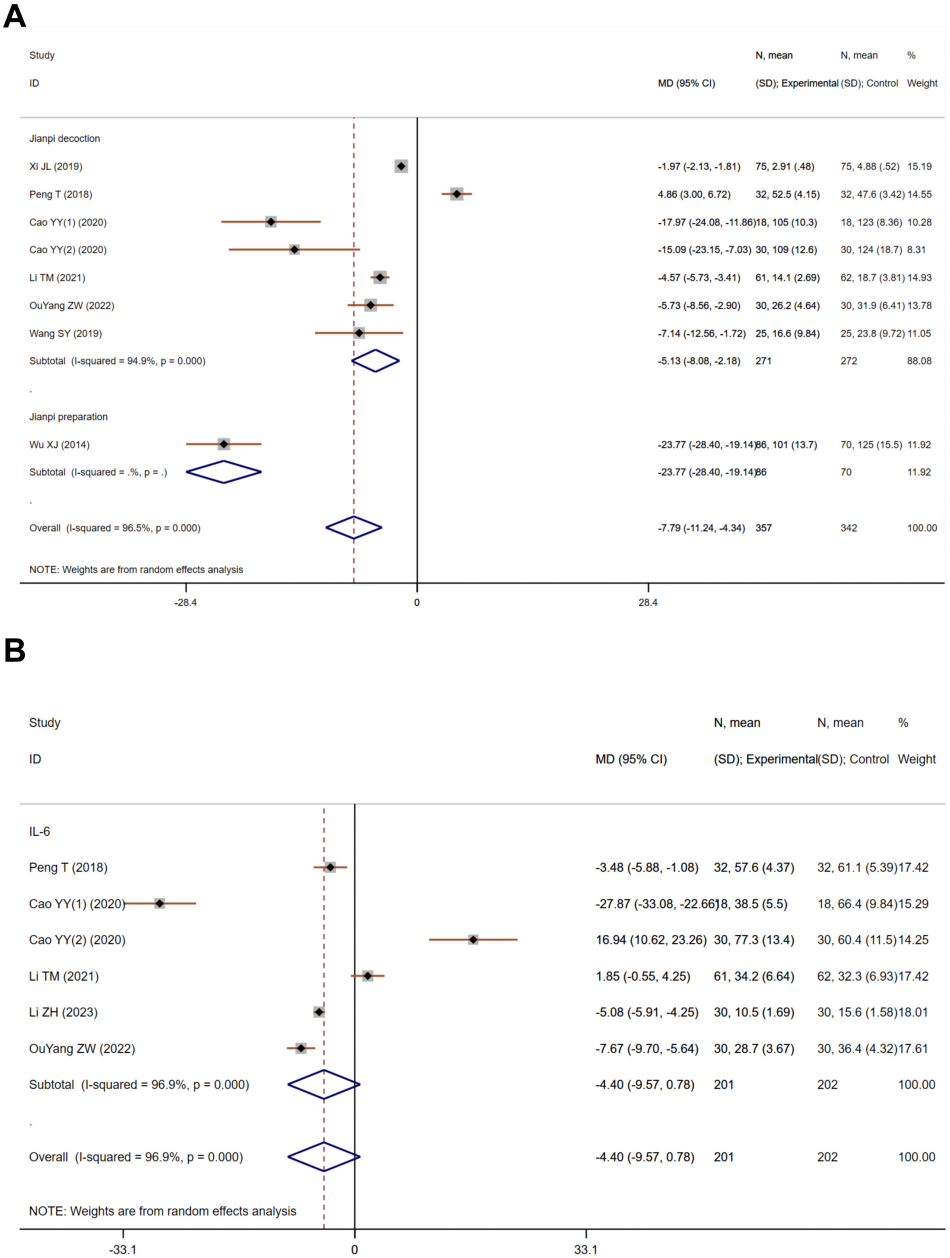
The effect of Jianpi therapy on TNF-α **(A)** and IL-6 **(B)** in CRF.

Tumor necrosis factor-alpha (TNF-α) and interleukin-6 (IL-6) are pivotal in immune and inflammatory responses, with chronic inflammation linked to fatigue. Cancer and its treatments can provoke the release of these pro-inflammatory cytokines, potentially leading to fatigue. Eight studies on TNF-α, totaling 699 participants—357 in the treatment and 342 in the control—showed significant heterogeneity (Heterogeneity test: Chi^2^ = 202.08, P<0.001, I^2^ = 96.5%). The pooled MD using a random-effects model was -7.79 (95% CI -11.24 to -4.34), indicating a significant reduction in TNF-α levels in the treatment group (Z=4.43, P<0.001, **Figure 4A**). Six studies on IL-6, including 403 participants—201 in the treatment and 202 in the control—also exhibited substantial heterogeneity (Heterogeneity test: Chi^2^ = 159.68, P<0.001, I^2^ = 96.9%). The pooled MD was -4.40 (95% CI -9.57 to 0.78), yet this did not reach statistical significance (Z=1.67, P=0.096, **Figure 4B**).

**Figure 5:**
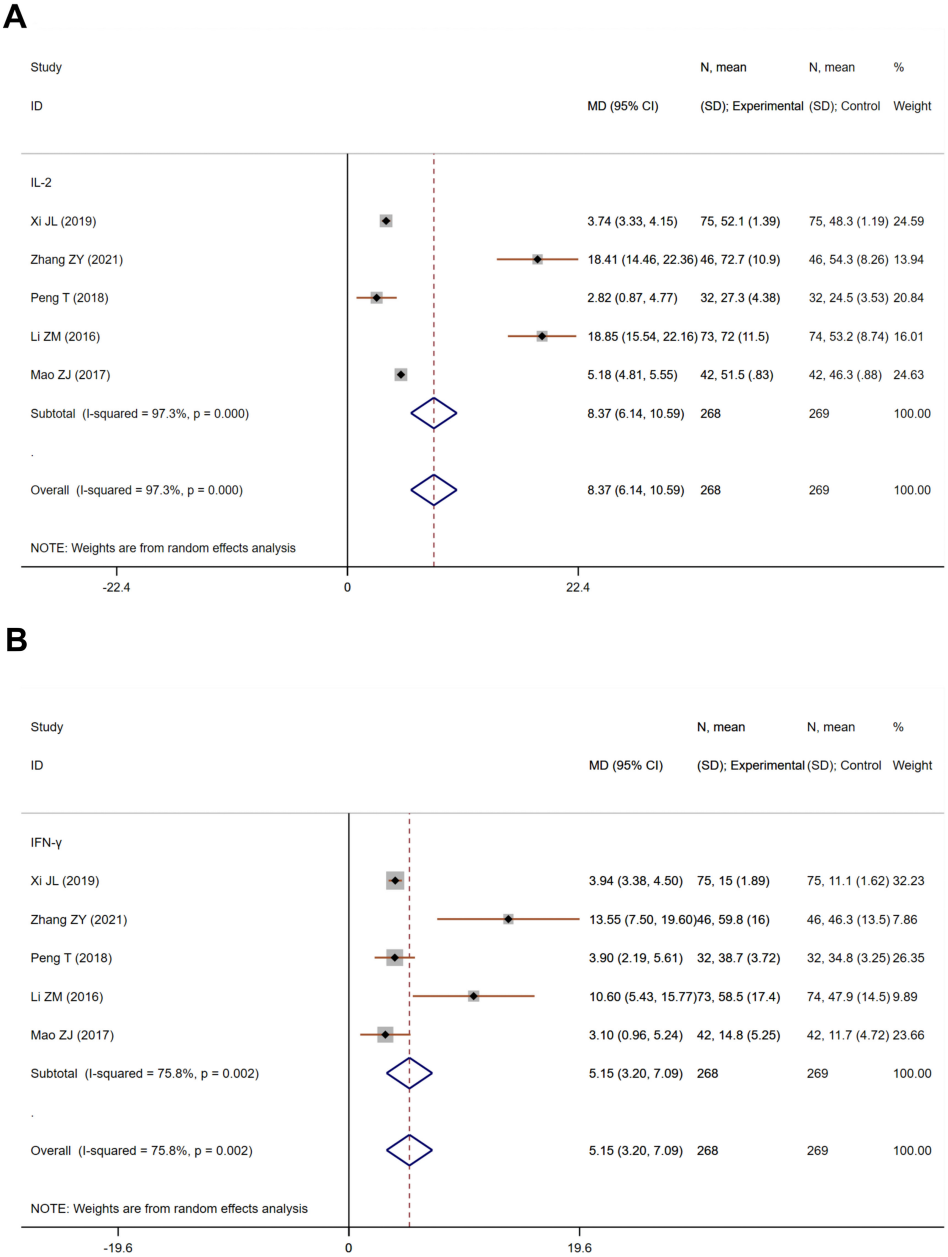
The effect of Jianpi therapy on IL-2 **(A)** and IFN-γ **(B)** in CRF.

Interleukin-2 (IL-2) and Interferon-γ (IFN-γ) are crucial for bolstering immune responses against cancer cells, with therapeutic applications in certain malignancies. Five studies on IL-2, encompassing 537 participants—268 in the treatment and 269 in the control—demonstrated considerable heterogeneity (Heterogeneity test: Chi^2^ = 147.28, P<0.001, I^2^ = 97.3%). The aggregated MD using a random-effects model was 8.37 (95% CI 6.14 to 10.59), signifying a marked increase in IL-2 levels in the treatment group (Z=7.37, P<0.001, **Figure 5A**). Five studies on IFN-γ, involving 537 participants—268 in the treatment and 269 in the control—also showed substantial heterogeneity (Heterogeneity test: Chi^2^ = 16.56, P=0.002, I^2^ = 75.8%). The pooled MD was 5.15 (95% CI 3.20 to 7.09), indicating a significant enhancement in IFN-γ levels in the treatment group (Z=5.19, P<0.001, **Figure 5B**).

#### 3.3.4 Impact of spleen-strengthening therapy on immune function

Cancer can establish an immunosuppressive milieu, impairing T cell function and potentially leading to immune exhaustion, associated with fatigue. CD3, CD4, and CD8 are surface markers on T cells, critical for immune responses. Fifteen studies on CD3 proportion, including 1,227 participants—617 in the treatment and 610 in the control—exhibited substantial heterogeneity (Heterogeneity test: Chi^2^ = 322.90, P<0.001, I^2^ = 95.7%). The random-effects model aggregated SMD was 0.84 (95% CI 0.23 to 1.44), indicating a significant improvement in CD3 proportion in the treatment group (Z=2.71, P=0.007, **Supplementary Figure 1A**). Subgroup analysis highlighted combined therapy as most effective for enhancing CD3 proportion (SMD=1.25, 95% CI 0.46 to 2.04, Z=3.09, P<0.001, I^2^ = 93.2%). No publication bias was detected by Egger’s test (P=0.580, **Supplementary Figure 1B**). Eighteen studies on CD4 proportion, totaling 1,511 participants—756 in the treatment and 755 in the control—also demonstrated substantial heterogeneity (Heterogeneity test: Chi^2^ = 355.99, P<0.001, I^2^ = 95.2%). The pooled SMD was 1.22 (95% CI 0.69 to 1.75), suggesting a significant improvement in CD4 proportion in the treatment group (Z=4.53, P<0.001, **Supplementary Figure 2A**). Subgroup analysis identified Jianpi decoction as most beneficial for CD4 proportion enhancement (SMD=1.34, 95% CI 0.54 to 2.31, Z=3.30, P=0.001, I^2^ = 96.5%). No publication bias was evident by Egger’s test (P=0.057, **Supplementary Figure 2B**). 17 studies on CD8 proportion, involving 1,349 participants—675 in the treatment and 674 in the control—showed substantial heterogeneity (Heterogeneity test: Chi^2^ = 324.05, P<0.001, I^2^ = 95.1%). The pooled SMD was -0.22 (95% CI -0.72 to 0.29), indicating no significant change in CD8 proportion between treatment and control groups (Z=0.84, P=0.403, **Supplementary Figure 3A**). No publication bias was detected by Egger’s test (P=0.937, **Supplementary Figure 3B**).

### 3.4 Sensitivity analysis

**Figure 6:**
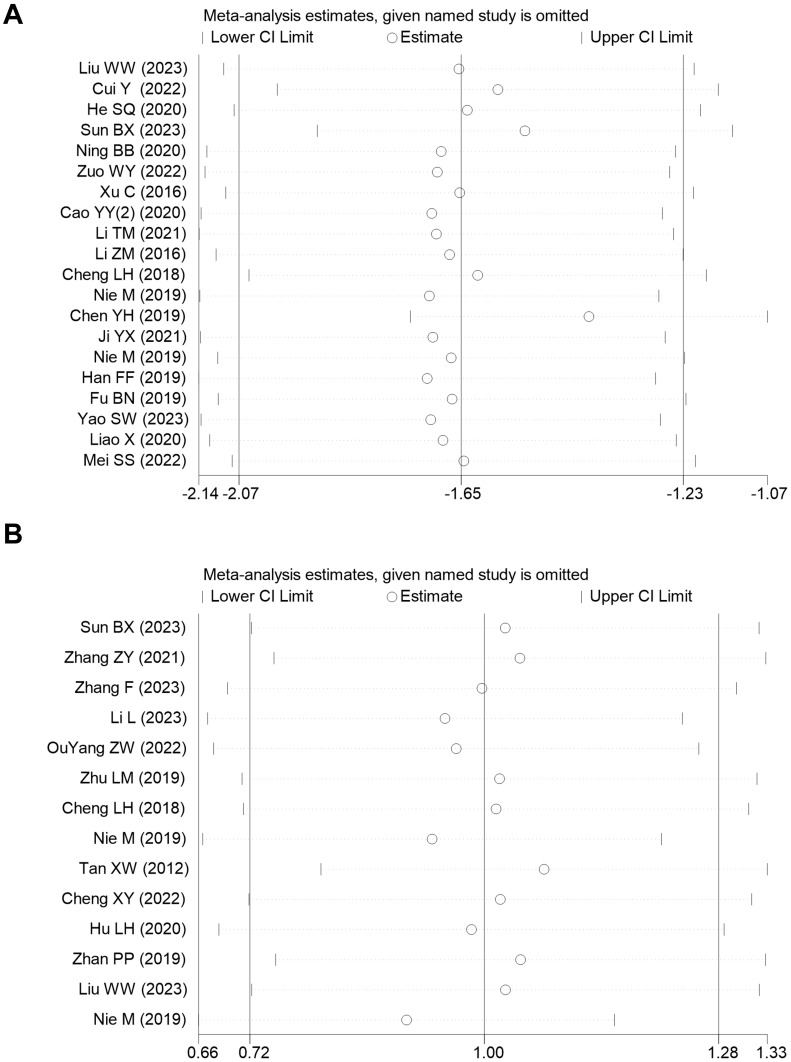
**(A)** Sensitivity analysis of PFS in CRF in the meta-analysis; **(B)** Sensitivity analysis of Hb in CRF in the meta-analysis.

**Figure 7:**
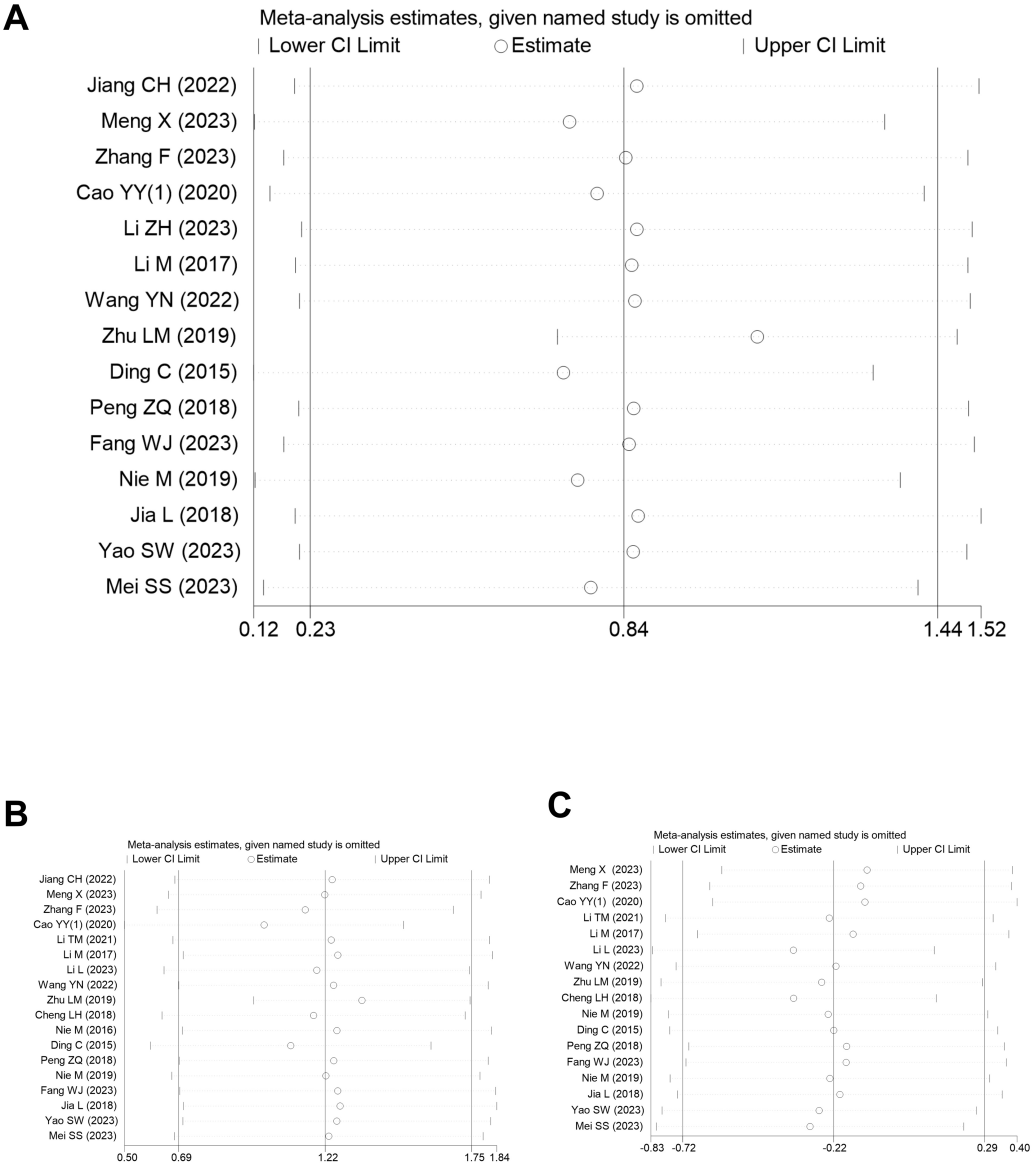
**(A)** Sensitivity analysis of CD3 in CRF in the meta-analysis; **(B)** Sensitivity analysis of CD4 in CRF in the meta-analysis; **(C)** Sensitivity analysis of CD8 in CRF in the meta-analysis.

Sensitivity analyses, conducted by sequentially excluding individual studies, were employed to assess the influence of each study on the pooled outcomes. The results demonstrated that the stability of the outcomes remained intact (**Figures 6**, **7**), thereby substantiating the reliability and robustness of our analytical approach.

### 3.5 Risk of bias assessment

**Figure 8:**
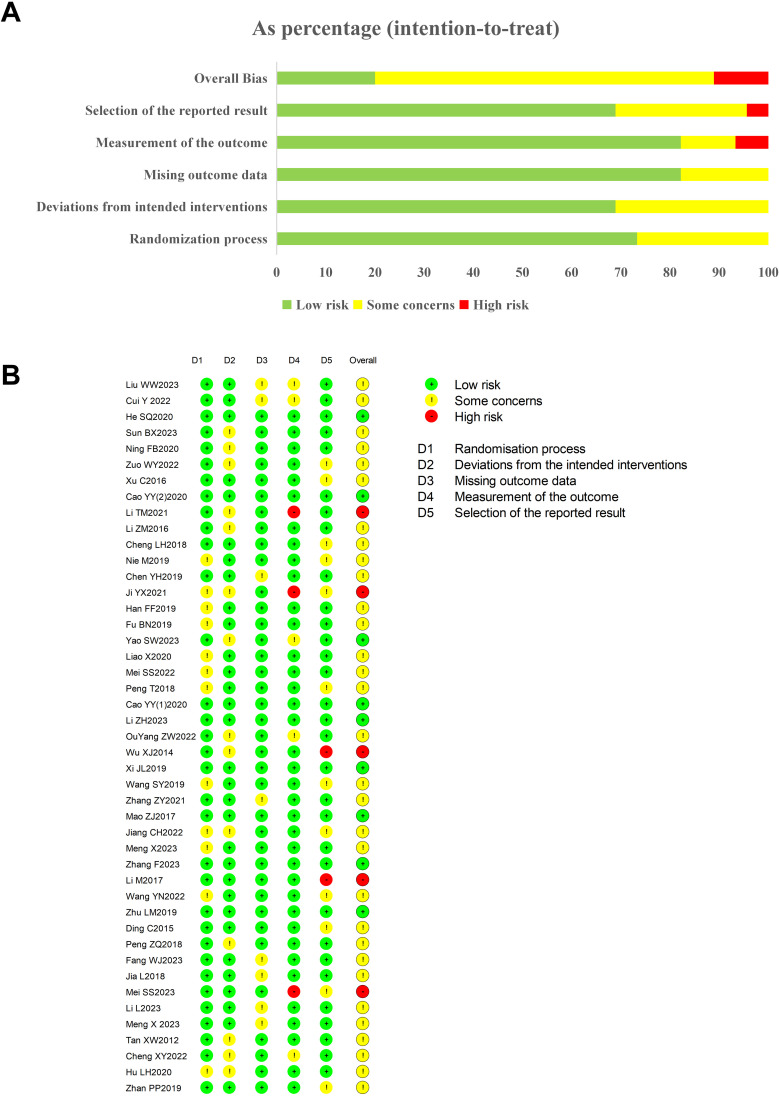
**(A, B)** Risk of bias summary of included studies.

Risk of bias was evaluated using the RoB 2.0 tool. Thirty-three studies employed random allocation with no discernible baseline disparities, suggesting a low risk of bias in randomization. Fourteen trials incorporated placebo-controlled, double-blind designs; the rest lacked blinding, raising concerns about adherence to intervention protocols. Thirty-seven studies reported comprehensive data, indicating a low risk of bias in data completeness. Three studies did not utilize objective outcome measures, presenting a higher risk of bias in outcome assessment. Two studies were deemed high risk due to inadequate methods for outcome data measurement, while 12 studies raised concerns about selective reporting, lacking a solid foundation for outcome assessment. These findings offer a comprehensive risk of bias evaluation, underscoring the strengths and vulnerabilities in the methodological approaches of the included studies (**Figure 8**).

### 3.6 Certainty of evidence

The evidence was graded based on criteria such as risk of bias, confidence intervals, and trial consistency. IL-2, TNF-α, IL-6, IFN-γ, CD3, CD4, and CD8 were assigned a moderate quality rating, whereas PFS and hemoglobin were given a low-quality rating (**Supplementary Table 5**). These assessments reflect the varying levels of confidence in the findings across different outcomes, pinpointing areas that may benefit from further research to bolster the evidence.

### 3.7 TSA

The TSA boundary plot, illustrating the effect of Jianpi therapy on the PFS, was constructed using the required information size (RIS), as shown **Supplementary Figure 4**. The RIS for this analysis was calculated to be 424, representing the number of participants needed to achieve a statistically reliable result with adequate power. In the plot, the cumulative Z-curve crosses both the conventional significance boundary (Z = 1.96) and the RIS boundary. The conventional boundary indicates statistical significance (p-value of 0.05), meaning the observed effect is unlikely to be due to chance. However, the RIS boundary is more critical, as it represents the point where enough data has been accumulated to provide a reliable estimate of the treatment effect. The crossing of both boundaries at the same point (RIS = 424) suggests that, at this stage, the evidence for the effectiveness of the Jianpi therapy is statistically significant and robust, making the possibility of a false-positive result unlikely. This indicates that the observed reduction in CRF, as measured by the PFS, is a true effect. Moreover, since the RIS boundary has been crossed, further data collection would not significantly alter the conclusion, confirming that the intervention’s effectiveness is reliable based on the current sample size.

## 4 Discussions

In TCM, the spleen is attributed with the critical functions of digestion and absorption, and it is pivotal in the generation of Qi, the life force, and blood (73). A deficiency in the spleen’s strength can manifest as symptoms such as lethargy, diminished appetite, and limb weakness, which are commonly associated with conditions like CRF. The etiology of CRF from the TCM perspective may encompass various elements, including a weakened spleen, stagnation of the liver, and debility of the kidneys (74). These factors can result in a depletion of Qi and blood, leading to the onset of fatigue. Consequently, therapeutic interventions are directed towards fortifying the spleen to enhance its transformative capabilities, enriching the blood, and bolstering the individual’s overall vitality (75). The spleen has been demonstrated to exert a substantial influence on the progression and metastasis of cancer by modulating immune responses. Two studies have demonstrated that splenic myeloid-derived suppressor cells and regulatory T cells inhibit CD8+ T cell responses, promote tumor growth, and result in a poorer prognosis and fatigue in cancer patients (76, 77).

This study suggests that interventions aimed at strengthening the spleen can reduce physical exhaustion by increasing hemoglobin levels. A strong association exists between anemia and the incidence of fatigue, particularly in cases of severe anemia, where individuals with anemia display a more pronounced frequency of fatigue-related symptoms (78). The decrease in hemoglobin levels due to anemia adversely affects the blood’s ability to transport oxygen, which in turn negatively impacts the oxygenation of tissues and cells. Given that oxygen is essential for cellular energy production, hypoxia can disrupt nutrient metabolism and consequently induce fatigue, whereas increases in hemoglobin levels have been shown to correlate with reduced fatigue and improvements in physical strength, functional capabilities, emotional stability, and overall health (79, 80). Further subgroup analysis indicates that the application of spleen-tonifying plasters significantly boosts hemoglobin levels, thereby enhancing oxygen delivery and reducing fatigue. In contrast to other methods of Jianpi therapy, TCM plasters present several benefits, including higher drug concentrations, improved bioavailability, a mild and sustained therapeutic impact, ease of use, and better taste, all of which contribute to improved patient compliance. Moreover, the viscosity of the plaster and its gradual absorption rate extend its duration of action, thereby amplifying its nourishing and curative properties (81).

The results of our research indicate that interventions designed to tonify the spleen can lead to a significant decrease in circulating TNF-α levels. This offers a potential mechanism through which these approaches may alleviate fatigue by attenuating systemic inflammation. Inflammation is a critical mechanism in the development of cancer-related fatigue, and TNF-α plays a vital role in the development and exacerbation of the syndrome (6, 82). TNF-α has been shown to stimulate cyclooxygenase activity, increasing prostaglandin production, including the vasodilator Prostaglandin H2 (PGH2), a hallmark of inflammation (83, 84). Furthermore, it exacerbates oxidative stress in inflamed regions and contributes to the induction of fever (85–87). A study by Garcia-Gonzalez et al. found that inflammation, the hypothalamic-pituitary-adrenal axis, nervous system and diet can all cause cancer-related fatigue in breast cancer survivors (88). Despite the absence of significant IL-6 reduction, possibly due to the limited scope and quality of the studies analyzed, our results also point to elevated levels of IFN-γ and IL-2 following spleen-tonifying treatment. IFN-γ, a multifaceted cytokine secreted by cells, regulates the maturation and activation of immune cells (89, 90). In a pioneering study, Alsbach and colleagues advanced the hypothesis that IFN-γ exerts an antitumor effect by impeding the proliferation of tumor cells, stimulating the activation of myeloid cells, promoting antigen presentation, and helping to clear tumor cells through the activation of tumor-specific immune responses, such as enhancing the cytotoxic activity of CD8+ T cells (91). IFN-γ is closely associated with the onset of cancer-related fatigue. IFN-γ induces systemic inflammatory responses by increasing the levels of IL-6 and TNF-α, which lead to changes in energy metabolism and neural function, resulting in fatigue symptoms in cancer patients (6). IL-2 has an important role in enhancing antitumor immune responses. Kamimura and Bevan studied the *in vivo* effect of IL-2 signaling on naive CD8+ T cells, finding that IL-2 and anti-IL-2 complexes alone triggered extensive T cell proliferation and differentiation into functional memory cells with a central memory phenotype (92). It stimulates the proliferation and activation of CD8+ T cells and NK cells, directly boosting immune-mediated tumor destruction (93). Similar to the role of IFN-γ, IL-2 has also been demonstrated to play a significant role in the development of cancer-related fatigue. In some cancer patients, IL-2 may induce excessive immune activation, leading to systemic inflammatory responses and fatigue. Particularly during immunotherapy, the overactivation of the immune system and subsequent cytokine release can exacerbate fatigue symptoms (94). Our findings suggest that spleen-tonifying preparations may selectively enhance CD3 and CD4 or CD8 cells, indicating their distinct advantages in immunomodulation. In the treatment of cancer, the efficacy of antitumor immunity is predominantly associated with the functionality of CD3+ T cells, which initiate the immune response by recognizing tumor-associated antigens, and CD4+ T cells, which primarily function through the paracrine immune response by activating CD8+ T cells and enhancing the antitumor immune response (95). In subgroup analysis, combined therapy most notably promoted CD3 and CD4 cells, while the promotion of CD8 cells was more pronounced, indicating that different TCM spleen-strengthening preparations have their unique benefits. Combined therapy encompasses traditional Chinese medicine spleen-strengthening prescriptions, Western medicine essential treatments, emotional therapy, and other treatment modalities. It addresses cancer fatigue from various perspectives and offers diverse approaches and strategies for its management. Compared with decoctions and oral Chinese patent medicines, spleen-strengthening preparations have the advantages of a singular composition, straightforward differentiation, and avoidance of gastrointestinal irritation (96).

In this meta-analysis, significant heterogeneity was observed in the efficacy of Jianpi therapy in the treatment of CRF. Potential causes for this heterogeneity may include, but are not limited to, differences in study design, patient populations, and intervention strategies. Jianpi therapy, a traditional Chinese medicinal treatment, is typically comprised of a combination of herbal formulas, acupuncture, and other modalities. These treatments exhibit significant differences, particularly in drug selection, dosage, duration, and frequency. These variations may contribute to the observed heterogeneity in study outcomes. In this analysis, a range of Jianpi herbal formulas (e.g., Sijunzi Tang and Buzhong Yiqi Tang), different treatment durations and frequencies, and individualized treatment approaches were used (18). A meta-analysis of 57 studies with 34,310 participants demonstrated significant variations in the prevalence and severity of CRF according to tumor progression, treatment stage, treatment modality, and gender (97). This heterogeneity may, stem from the differences in patients’ backgrounds in the current study, which did not differentiate and refine factors such as patients’ cancer type and treatment stage. The final and most significant rationale pertains to the distinct characteristics of Chinese medicine treatment, which, in contrast to the universal adaptability of modern medical diagnosis and treatment, emphasizes an individualized approach to diagnosis and treatment. In a seminal study, Sumei Wang and colleagues advanced the notion of employing TCM as a personalized therapeutic modality in the context of human cancer. This proposition signifies a potentially efficacious strategy for addressing cancerous afflictions in humans (98). This approach acknowledges that patients’ constitution may exhibit variations, resulting in divergent treatment outcomes. Patients may combine different TCM evidence types, such as weak Qi and blood, weak spleen and stomach, which may affect their response to Jianpi therapy. Consequently, the efficacy of Jianpi therapy may vary significantly among patients, with some demonstrating a more favorable response compared to others.

Several potential limitations must be acknowledged. To start with, the results of the Jianpi therapy for CRF demonstrated notable heterogeneity, which underscores the study’s limitations. This variability may potentially affect the generalizability of the findings to a broader group of cancer patients. Moreover, the absence of a standardized treatment for CRF results in diverse treatment modalities and therapeutic effects, introducing bias into our research findings. Finally, due to data limitations, there may be additional spleen-tonifying therapies that we could potentially collect.

## 5 Conclusions

The current meta-analysis concludes that Jianpi therapy could significantly alleviate symptoms in patients with CRF. Thus, Jianpi therapy may be an effective treatment for CRF. The results underscore the efficacy of traditional Chinese medicine in managing CRF.

## Data Availability

The original contributions presented in the study are included in the article/**Supplementary Material**. Further inquiries can be directed to the corresponding author.
