# Supplementary material for: Effects of Jianpi therapy for cancer-related fatigue:a meta-analysis of randomized controlled trials
Source: Front Oncol. 2025 Jan 24;15:1512460. doi: 10.3389/fonc.2025.1512460 (PMC11840261; doi:10.3389/fonc.2025.1512460)
Supplement: Supplementary file 1 [file DataSheet1.docx]

Supplementary Material

Supplementary Table 1 Search strategies

| **PubMed** |
| --- |
| ("cancer related fatigue"[Title/Abstract] OR "tumor related fatigue"[Title/Abstract] OR "fatigue syndrome chronic"[Title/Abstract] OR "fatigue"[Title/Abstract] OR "CRF"[Title/Abstract] OR "asthenia"[Title/Abstract] OR "tired"[Title/Abstract] OR "weary"[Title/Abstract] OR "weariness"[Title/Abstract] OR "exhaust*"[Title/Abstract] OR "asthenia"[Title/Abstract] OR "asthenic"[Title/Abstract] OR "lassitude"[Title/Abstract] OR "lethargy"[Title/Abstract] OR "exhaustion"[Title/Abstract] OR "tiredness"[Title/Abstract]) AND ("medicine chinese traditional"[Title/Abstract] OR "traditional chinese medicine"[Title/Abstract] OR "traditional chinese herbal formula"[Title/Abstract] OR "chinese herbal medicine"[Title/Abstract] OR "herbal formula"[Title/Abstract] OR "herbs"[Title/Abstract] OR "alternative medicine"[Title/Abstract] OR "panax"[Title/Abstract] OR "poria"[Title/Abstract] OR "glycyrrhiza uralensis"[Title/Abstract] OR "huang qi"[Title/Abstract] OR "rhizoma atractylodis"[Title/Abstract] OR "atractylodes macrocephala"[Title/Abstract] OR "radix angelicae sinensis"[Title/Abstract] OR "semen ziziphi spinosae"[Title/Abstract] OR "radix codonopsis pilosulae"[Title/Abstract] OR "strengthening spleen"[Title/Abstract] OR "replenishing spleen"[Title/Abstract] OR "invigorating spleen"[Title/Abstract] OR "Jianpi"[Title/Abstract]) |
| **EMbase** |
| 'medicine chinese traditional':ab,ti OR 'traditional chinese medicine':ab,ti OR 'traditional chinese herbal formula':ab,ti OR 'chinese herbal medicine':ab,ti OR 'herbal formula':ab,ti OR 'herbs':ab,ti OR 'alternative medicine':ab,ti OR 'panax':ab,ti OR 'poria':ab,ti OR 'glycyrrhiza uralensis':ab,ti OR 'huang qi':ab,ti OR 'rhizoma atractylodis':ab,ti OR 'atractylodes macrocephala':ab,ti OR 'radix angelicae sinensis':ab,ti OR 'semen ziziphi spinosae':ab,ti OR 'radix codonopsis pilosulae':ab,ti OR 'strengthening spleen':ab,ti OR 'replenishing spleen':ab,ti OR 'invigorating spleen':ab,ti OR 'jianpi':ab,ti |
| **Cochrane Library** |
| (medicine chinese traditional):ab,ti,kw OR (traditional chinese medicine):ab,ti,kw OR (traditional chinese herbal formula):ab,ti,kw OR (chinese herbal medicine):ab,ti,kw OR (herbal formula):ab,ti,kw OR (herbs):ab,ti,kw OR (alternative medicine):ab,ti,kw OR (panax):ab,ti,kw OR (poria):ab,ti,kw OR (glycyrrhiza uralensis):ab,ti,kw OR (huang qi):ab,ti,kw OR (rhizoma atractylodis):ab,ti,kw OR (atractylodes macrocephala):ab,ti,kw OR (radix angelicae sinensis):ab,ti,kw OR (semen ziziphi spinosae):ab,ti,kw OR (radix codonopsis pilosulae):ab,ti,kw OR (strengthening spleen):ab,ti,kw OR (replenishing spleen):ab,ti,kw OR (invigorating spleen):ab,ti,kw OR (jianpi):ab,ti,kw |
| **Web of Science** |
| TS=(medicine chinese traditional OR traditional chinese medicine OR traditional chinese herbal formula OR chinese herbal medicine OR herbal formula OR herbs OR alternative medicine OR panax OR poria OR glycyrrhiza uralensis OR huang qi OR rhizoma atractylodis OR atractylodes macrocephala OR radix angelicae sinensis OR semen ziziphi spinosae OR radix codonopsis pilosulae OR strengthening spleen OR replenishing spleen OR invigorating spleen OR Jianpi) |
| Sinomed |
| ("健脾"[标题:智能] OR "补脾"[标题:智能] OR "运脾"[标题:智能] OR "培土"[标题:智能] OR "补土"[标题:智能] OR "益脾"[标题:智能] OR "化湿"[标题:智能] OR "祛湿"[标题:智能]) AND ("癌性疲乏"[标题:智能] OR "癌性疲劳"[标题:智能] OR "癌因性疲乏"[标题:智能] OR "癌相关性乏力"[标题:智能] OR "疲惫"[标题:智能] OR "疲乏"[标题:智能] OR "乏力"[标题:智能] OR "疲劳"[标题:智能] OR "疲倦"[标题:智能]) |
| Wanfang Data |
| 题名或关键词:(癌性疲乏 or 癌性疲乏 or 癌因性疲乏 or 癌相关性乏力 or 癌相关性疲劳 or 疲乏 or 乏力 or 疲劳 or 疲劳) and 题名或关键词:(健脾 or 补脾 or 运脾 or 培土 or 补土 or 益脾 or 益气健脾法 or 脾气虚 or 化湿 or 祛湿) |
| China National Knowledge Infrastructure |
| TI=(癌性疲乏 OR 癌性疲乏 OR 癌因性疲乏 OR 癌相关性乏力 OR 癌相关性疲劳 OR 疲乏 OR 乏力 OR 疲劳 OR 疲劳) AND (健脾 OR 补脾 OR 运脾 OR 培土 OR 补土 OR 益脾 OR 益气健脾法 OR 脾气虚 OR 化湿 OR 祛湿) |
| China Science and Technology Journal Database |
| M=(癌性疲乏 OR 癌性疲乏 OR 癌因性疲乏 OR 癌相关性乏力 OR 癌相关性疲劳 OR 疲乏 OR 乏力 OR 疲劳 OR 疲劳) AND M=(健脾 OR 补脾 OR 运脾 OR 培土 OR 补土 OR 益脾 OR 益气健脾法 OR 脾气虚 OR 化湿 OR 祛湿) |

Supplementary Table 2: PRISMA 2020 checklist

| **Section and Topic** | **Item #** | **Checklist item** | **Location where item is reported** |
| --- | --- | --- | --- |
| **TITLE** | | |  |
| Title | 1 | Identify the report as a systematic review. | 1 |
| **ABSTRACT** | | |  |
| Abstract | 2 | See the PRISMA 2020 for Abstracts checklist. | 1 |
| **INTRODUCTION** | | |  |
| Rationale | 3 | Describe the rationale for the review in the context of existing knowledge. | 2 |
| Objectives | 4 | Provide an explicit statement of the objective(s) or question(s) the review addresses. | 2 |
| **METHODS** | | |  |
| Eligibility criteria | 5 | Specify the inclusion and exclusion criteria for the review and how studies were grouped for the syntheses. | 3 |
| Information sources | 6 | Specify all databases, registers, websites, organisations, reference lists and other sources searched or consulted to identify studies. Specify the date when each source was last searched or consulted. | 3 |
| Search strategy | 7 | Present the full search strategies for all databases, registers and websites, including any filters and limits used. | 3 |
| Selection process | 8 | Specify the methods used to decide whether a study met the inclusion criteria of the review, including how many reviewers screened each record and each report retrieved, whether they worked independently, and if applicable, details of automation tools used in the process. | S1 |
| Data collection process | 9 | Specify the methods used to collect data from reports, including how many reviewers collected data from each report, whether they worked independently, any processes for obtaining or confirming data from study investigators, and if applicable, details of automation tools used in the process. | 3 |
| Data items | 10a | List and define all outcomes for which data were sought. Specify whether all results that were compatible with each outcome domain in each study were sought (e.g. for all measures, time points, analyses), and if not, the methods used to decide which results to collect. | 3 |
|  | 10b | List and define all other variables for which data were sought (e.g. participant and intervention characteristics, funding sources). Describe any assumptions made about any missing or unclear information. | 3 |
| Study risk of bias assessment | 11 | Specify the methods used to assess risk of bias in the included studies, including details of the tool(s) used, how many reviewers assessed each study and whether they worked independently, and if applicable, details of automation tools used in the process. | 3 |
| Effect measures | 12 | Specify for each outcome the effect measure(s) (e.g. risk ratio, mean difference) used in the synthesis or presentation of results. | 3 |
| Synthesis methods | 13a | Describe the processes used to decide which studies were eligible for each synthesis (e.g. tabulating the study intervention characteristics and comparing against the planned groups for each synthesis (item #5)). | 3 |
|  | 13b | Describe any methods required to prepare the data for presentation or synthesis, such as handling of missing summary statistics, or data conversions. | 4 |
|  | 13c | Describe any methods used to tabulate or visually display results of individual studies and syntheses. | 4 |
|  | 13d | Describe any methods used to synthesize results and provide a rationale for the choice(s). If meta-analysis was performed, describe the model(s), method(s) to identify the presence and extent of statistical heterogeneity, and software package(s) used. | 4 |
|  | 13e | Describe any methods used to explore possible causes of heterogeneity among study results (e.g. subgroup analysis, meta-regression). | 4 |
|  | 13f | Describe any sensitivity analyses conducted to assess robustness of the synthesized results. | 6 |
| Reporting bias assessment | 14 | Describe any methods used to assess risk of bias due to missing results in a synthesis (arising from reporting biases). | 6 |
| Certainty assessment | 15 | Describe any methods used to assess certainty (or confidence) in the body of evidence for an outcome. | 7 |
| **RESULTS** | | |  |
| Study selection | 16a | Describe the results of the search and selection process, from the number of records identified in the search to the number of studies included in the review, ideally using a flow diagram. | 4 |
|  | 16b | Cite studies that might appear to meet the inclusion criteria, but which were excluded, and explain why they were excluded. | 4 |
| Study characteristics | 17 | Cite each included study and present its characteristics. | 4 |
| Risk of bias in studies | 18 | Present assessments of risk of bias for each included study. | 6 |
| Results of individual studies | 19 | For all outcomes, present, for each study: (a) summary statistics for each group (where appropriate) and (b) an effect estimate and its precision (e.g. confidence/credible interval), ideally using structured tables or plots. | 5 |
| Results of syntheses | 20a | For each synthesis, briefly summarise the characteristics and risk of bias among contributing studies. | 5 |
|  | 20b | Present results of all statistical syntheses conducted. If meta-analysis was done, present for each the summary estimate and its precision (e.g. confidence/credible interval) and measures of statistical heterogeneity. If comparing groups, describe the direction of the effect. | 5 |
|  | 20c | Present results of all investigations of possible causes of heterogeneity among study results. | 5 |
|  | 20d | Present results of all sensitivity analyses conducted to assess the robustness of the synthesized results. | 6 |
| Reporting biases | 21 | Present assessments of risk of bias due to missing results (arising from reporting biases) for each synthesis assessed. | 6 |
| Certainty of evidence | 22 | Present assessments of certainty (or confidence) in the body of evidence for each outcome assessed. | 6 |
| **DISCUSSION** | | |  |
| Discussion | 23a | Provide a general interpretation of the results in the context of other evidence. | 7 |
|  | 23b | Discuss any limitations of the evidence included in the review. | 7 |
|  | 23c | Discuss any limitations of the review processes used. | 7 |
|  | 23d | Discuss implications of the results for practice, policy, and future research. | 8 |
| **OTHER INFORMATION** | | |  |
| Registration and protocol | 24a | Provide registration information for the review, including register name and registration number, or state that the review was not registered. | 2 |
|  | 24b | Indicate where the review protocol can be accessed, or state that a protocol was not prepared. | 2 |
|  | 24c | Describe and explain any amendments to information provided at registration or in the protocol. | 2 |
| Support | 25 | Describe sources of financial or non-financial support for the review, and the role of the funders or sponsors in the review. | 8 |
| Competing interests | 26 | Declare any competing interests of review authors. | 8 |
| Availability of data, code and other materials | 27 | Report which of the following are publicly available and where they can be found: template data collection forms; data extracted from included studies; data used for all analyses; analytic code; any other materials used in the review. | 8 |

Supplementary Table 3: PRISMA 2020 for abstracts checklist

| **Section and Topic** | **Item #** | **Checklist item** | **Reported (Yes/No)** |
| --- | --- | --- | --- |
| **TITLE** | | |  |
| Title | 1 | Identify the report as a systematic review. | YES |
| **BACKGROUND** | | |  |
| Objectives | 2 | Provide an explicit statement of the main objective(s) or question(s) the review addresses. | YES |
| **METHODS** | | |  |
| Eligibility criteria | 3 | Specify the inclusion and exclusion criteria for the review. | YES |
| Information sources | 4 | Specify the information sources (e.g. databases, registers) used to identify studies and the date when each was last searched. | YES |
| Risk of bias | 5 | Specify the methods used to assess risk of bias in the included studies. | YES |
| Synthesis of results | 6 | Specify the methods used to present and synthesise results. | YES |
| **RESULTS** | | |  |
| Included studies | 7 | Give the total number of included studies and participants and summarise relevant characteristics of studies. | YES |
| Synthesis of results | 8 | Present results for main outcomes, preferably indicating the number of included studies and participants for each. If meta-analysis was done, report the summary estimate and confidence/credible interval. If comparing groups, indicate the direction of the effect (i.e. which group is favoured). | YES |
| **DISCUSSION** | | |  |
| Limitations of evidence | 9 | Provide a brief summary of the limitations of the evidence included in the review (e.g. study risk of bias, inconsistency and imprecision). | YES |
| Interpretation | 10 | Provide a general interpretation of the results and important implications. | YES |
| **OTHER** | | |  |

Supplementary Table 4: Characteristics of included studies

| Study | No. of subjects | Control group intervention | Experimental group intervention | Criteria to assess 'CRF' | Treatment duration | Age,year |  |
| --- | --- | --- | --- | --- | --- | --- | --- |
| Liu WW2023   \|  \|  \| \| --- \| --- \| | 100 | placebo | Jianpi Fuzheng Decoction | ①② | 3w | 18-75 |  |
| Cui YX2022 | 94 | Placebo + chemotherapy | Astragalus Sijunzi Decoction + chemotherapy | ① | 20d | 18-69 |  |
| He SQ2020 | 68 | General treatment | General treatment + Spleen-invigorating kidney prescription | ①⑤ | 2w | 18 - 75 |  |
| Sun BX2023 | 100 | Non-drug intervention | Non-drug intervention + Bufei Jianpi Decoction | ①②⑤ | 4w | 18-75 |  |
| Ning BB2020 | 80 | Low dose of astragalus flavour-supplementing Zhongyiqi decoction | High dose of astragalus flavour-supplementing Zhongyiqi decoction | ①⑤ | 4w | 18-80 |  |
| Zuo WY2022 | 59 | Basic treatment + Guipi pills | Basic treatment + Guipi pills | ①② | 8w | 18-85 |  |
| Xu C2016 | 81 | Supportive treatment + symptomatic treatment | Jianpi Yishen formula | ① | 9w | 18-75 |  |
| Cao YY(2)2020 | 60 | Chemotherapy | Chemotherapy + flavored Sijunzi soup | ,①③⑤ | 2w | ＞18 |  |
| Li TM2021 | 123 | Supportive treatment | Spleen-yiqi formula | ①④⑤ | 4w | 18-80 |  |
| Li ZM2016 | 60 | DXM | DXM + Invigorating spleen and invigorating kidney traditional Chinese medicine | ① | 2w | ≥60 |  |
| Cheng LH2018 | 76 | Chemotherapy | Jianpi Bushen decoction | ①②④⑤ | 6w | 18-70 |  |
| Nie M2019 | 90 | Basic treatment | Basic treatment + Complement 1 square | ①②④⑤ | 2w | 18-70 |  |
| Chen YH2019 | 70 | Chemotherapy | Chemotherapy + Jianpi Xiaoji Decoction | ①⑤ | 40d | 40-65 |  |
| Ji YX2021 | 80 | Daily care | Daily care + mild moxibustion + Buzhong Yiqi decoction | ①⑤ | 2w | 18-80 |  |
| Han FF2019 | 72 | Sijunzi granule | Acupuncture + Moxibustion | ① | 12d | ＞18 |  |
| Fu BN2019 | 99 | Glucose injection + Xiaoaiping injection | Glucose injection + Xiaoaiping injection + Qiangshen Jianpi granule | ①②⑤ | 2w | 18-80 |  |
| Yao SW2023 | 50 | symptomatic treatment | symptomatic treatment + Qi GUI blood supplement syru | ①②④⑤ | 6w | 18-70 |  |
| Li X2020 | 66 | Basic treatment | Basic treatment + Shenqi Fuzheng injection | ① | 2w | 18-70 |  |
| Mei SS2022 | 30 | Non-drug treatment | Non-drug treatment + Buzhong Yiqi granules | ④⑤ | 30d | 18-80 |  |
| Peng T2018 | 64 | Chemotherapy | Chemotherapy + Yiqi Jianpi Quyu Huoxue formula | ④ | 8w | ＞18 |  |
| Cao YY(1)2020 | 36 | Chemotherapy | Chemotherapy + Yiqi Jianpi formula | ③④ | 10d | 18-70 |  |
| Li ZH2023 | 30 | Symptomatic treatment | Symptomatic treatment + Warming Yang tonifying kidney and spleen method | ③④⑤ | 14d | 18-75 |  |
| OuYang ZW2022 | 60 | Basic treatment | Basic treatment + Jianpi Yangrong Decoction | ①③⑤ | 3m | ≥20 |  |
| Wu XJ2014 | 120 | Chemotherapy | Chemotherapy + Shenmai injection | ①③⑤ | 8w | ＞18 |  |
| Xi JL2019 | 150 | Chemotherapy | Chemotherapy + spleen-strengthening prescription | ④⑤ | 24w | 20-70 |  |
| Wang SY2019 | 50 | Chemotherapy | Chemotherapy + invigorating spleen and supplementing blood | ③⑤ | 6w | ＞18 |  |
| Zhang ZY2021 | 92 | Chemotherapy | Chemotherapy + Spleen tonifying kidney tonifying blood decoction | ①⑤ | 1m | 38-50 |  |
| Mao ZJ2017 | 84 | Chemotherapy | Chemotherapy + spleen strengthening method | ④⑤ | 12w | 30-75 |  |
| Jiang CH2022 | 102 | Chemotherapy | Chemotherapy + Shengjin Decoction | ①④⑤ | 6w | 45-79 |  |
| Meng X2023 | 112 | Chemotherapy + Peiyuan Yiqi decoction | Chemotherapy + Peiyuan Yiqi decoction | ①④⑤ | 9w | 28-75 |  |
| Zhang F2023 | 100 | Chemotherapy + Shugan Jianpi formula | Chemotherapy + Shugan Jianpi formula | ①③④⑤ | 2w | 30-70 |  |
| Li M2017 | 64 | Chemotherapy | Chemotherapy + Invigorating spleen and hydrating water | ④ | 9w | 25-65 |  |
| Zhu LM2019 | 139 | Chemotherapy | Chemotherapy + Spleen-yishen-Jiedu prescription | ①④⑤ | 8w | 18-70 |  |
| Ding C2015 | 60 | Chemotherapy | Chemotherapy + Yiqi Jianpi decoction for internal administration fumigation | ④⑤ | 15d | 26-79 |  |
| Peng ZQ2018 | 60 | Conventional therapy | Yiqi Jianpi Gubenfang + thymus pentapeptide point injection | ④⑤ | 4w | 35-72 |  |
| Jia L2018 | 108 | Chemotherapy + intravenous infusion of glucopeptide + aerobic exercise | Chemotherapy + Spleen-yishen-Jiedu prescription + aerobic exercise | ④⑤ | 12w | ＜80 |  |
| Fang WJ2023 | 118 | Conventional therapy | Conventional therapy + wheat moxibustion combined Yiqi Jianpi formula | ④⑤ | 3m | 18-75 |  |
| Li L2023 | 84 | Chemotherapy | Chemotherapy + tonifying spleen and kidney method | ①②④ | 3w | 44-64 |  |
| Tan XW2012 | 63 | Chemotherapy | Chemotherapy + Jianpi Yiqi Huatan formula | ② | 2w | 18-70 |  |
| Cheng XY2022 | 72 | Basic treatment + Fuzheng Yiqi formula | Basic treatment + Fuzheng Yiqi formula | ①②⑤ | 2w | 18-80 |  |
| Zhan PP2019 | 80 | Chemotherapy | Chemotherapy + Jianpi Sheng pulp cream | ②⑤ | 6w | 18-75 |  |
| Wang YN2022 | 60 | Basic treatment | Basic treatment + Jianpi Fuzheng Decoction | ④⑤ | 4w | 18-75 |  |
| Mei SS2023 | 58 | non-medicine treatment | non-medicine treatment + Buzhong Yiqi granule | ④⑤ | 30d | 18-80 |  |
| Meng X2022 | 56 | Chemotherapy | Chemotherapy + Peiyuan Yiqi decoction | ①④⑤ | 63d | 28-75 |  |
| Hu LH2020 | 116 | Chemotherapy | Chemotherapy + Jianpi Shengpulp cream formula | ①②③⑤ | 20d | 18-75 |  |

**Notes: d:days, w:weeks**

①: **Piper Fatigue Scale (PFS)**

②: **Hemoglobin(Hb)**

③: **Cytokines**

④: **Immune Function**

⑤: **Others**

**▲: In order to distinguish two papers published by the same author in 2020, the included literatures were distinguished by (1) and (2).**

**Cao YY(1)2020: Cao Y, Zhu T. Effects of Yiqi Jianpi decoction on fatigue in malignant tumor patients after chemotherapy. Chin J Clin Res. 2020;3(08):1115-8.**

**Cao YY(2)2020: Cao Y. Clinical study on Jiawei Si Jun Zi decoction for treating cancer-related fatigue after chemotherapy. Liaoning J Tradit Chin Med. 2020;47(08):112-4.**

Supplementary Table 5: GRADE evidence profile

| Quality assessment | | | | | | No. of patients | MD/SMD  (95%CI) | P value | Quality |
| --- | --- | --- | --- | --- | --- | --- | --- | --- | --- |
| No. of studies | Risk of bias | Inconsistency | Indirectness | Imprecision | Other considerations |  |  |  |  |
| **IL-2 in CRF** | | | | | | | | | |
| 5 | no serious risk of bias | serious inconsistency ^a^ | no serious indirectness | no serious imprecision | none | 537 | 8.37  (6.14-10.59) | P =0.000 | ⊕⊕⊕O  MODERATE |
| **Piper fatigue scale in CRF** | | | | | | | | | |
| 20 | no serious risk of bias | serious inconsistency ^a^ | no serious indirectness | no serious imprecision | reporting bias ^b^ | 1,515 | -1.65  (-2.07-(-1.23)) | P =0.000 | ⊕⊕OO  LOW |
| **TNF-α in CRF** | | | | | | | | | |
| 8 | no serious risk of bias | serious inconsistency ^a^ | no serious indirectness | no serious imprecision | none | 699 | -7.79  (-11.24-(-4.34)) | P *=*0.000 | ⊕⊕⊕O  MODERATE |
| **Hemoglobin (Hb) in CRF** | | |  |  |  |  |  |  |  |
| 13 | no serious risk of bias | serious inconsistency ^a^ | no serious indirectness | no serious imprecision | reporting bias ^b^ | 1,173 | 8.64  (5.53-11.74) | P =0.000 | ⊕⊕OO  LOW |
| **Interleukin-6 (IL-6)** **in CRF** | | |  |  |  |  |  |  |  |
| 6 | no serious risk of bias | serious inconsistency ^a^ | no serious indirectness | no serious imprecision | none | 403 | -4.40  (-9.57-(-0.78)) | P=0.000 | ⊕⊕⊕O  MODERATE |
| **CD3 in CRF** | | | | |  |  |  |  |  |
| 15 | no serious risk of bias | serious inconsistency ^a^ | no serious indirectness | no serious imprecision | none | 1,127 | 0.84  (0.23-1.44) | P =0.000 | ⊕⊕⊕O  MODERATE |
| **IFN-γ in CRF** | | | |  |  |  |  |  |  |
| 5 | no serious risk of bias | serious inconsistency ^a^ | no serious indirectness | no serious imprecision | none | 537 | 5.15  (3.20-7.09) | P =0.002 | ⊕⊕⊕O  MODERATE |
| **CD4 in CRF** | | | | | |  |  |  |  |
| 18 | no serious risk of bias | serious inconsistency ^a^ | no serious indirectness | no serious imprecision | none | 1,511 | 1.22  (0.69-1.75) | P =0.000 | ⊕⊕⊕O  MODERATE |
| **CD8 in CRF** | | | |  |  |  |  |  |  |
| 16 | no serious risk of bias | serious inconsistency ^a^ | no serious indirectness | no serious imprecision | none | 1,349 | -0.22  (-0.72-0.29) | P =0.000 | ⊕⊕⊕O  MODERATE |
|  |  |  |  |  |  |  |  |  |  |

^a^ There is controversy in different studies. ^b^ Egger's test P＜0.05. RR rate ratio, SMD standard mean difference, CI confidence interval.

**Supplementary FIGURE :**

**
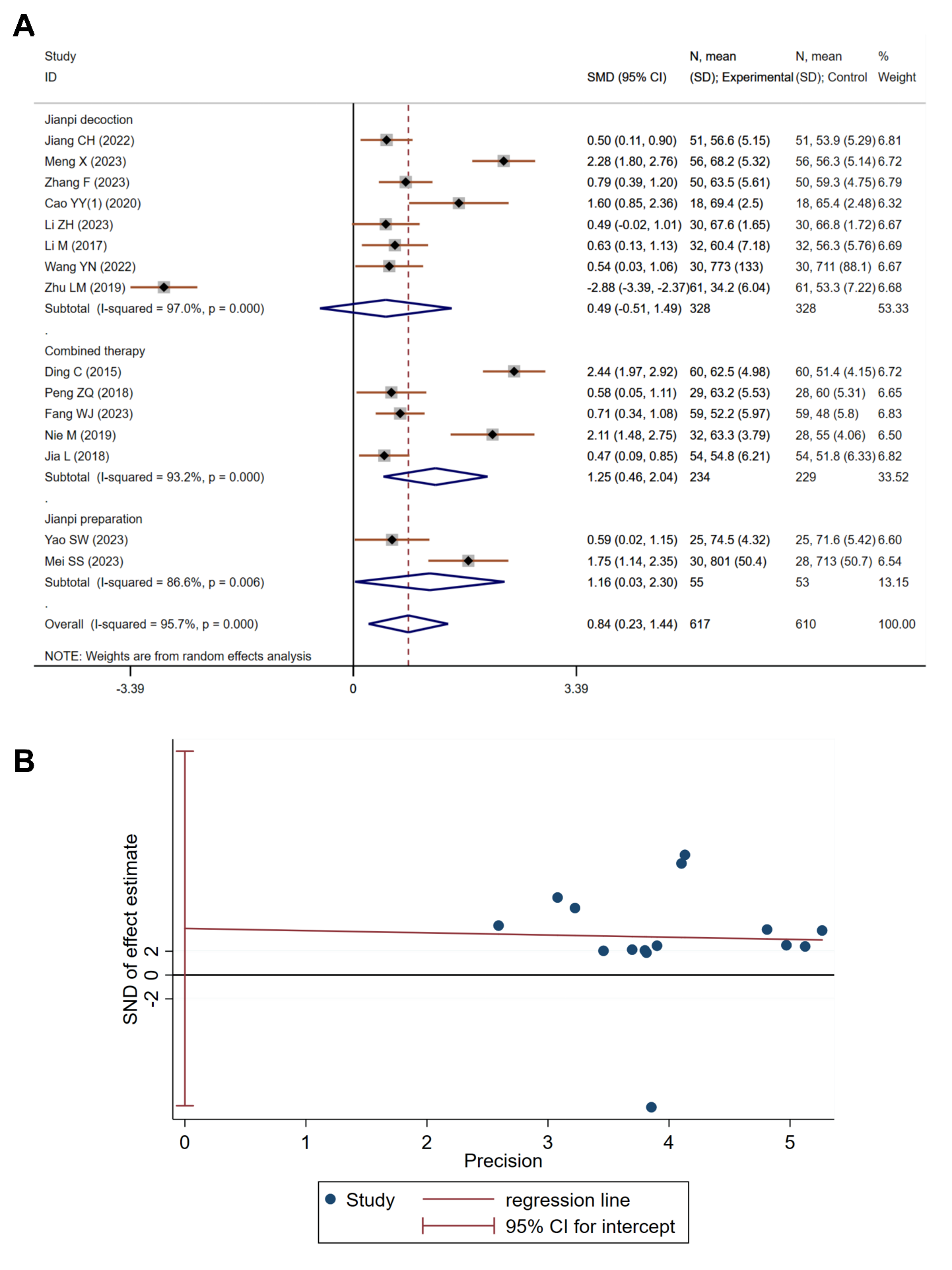
**

**Supplementary FIGURE 1. The effect of Jianpi therapy on CD3 in CRF. (A) Forrest plot. (B) Egger’s test.**

**
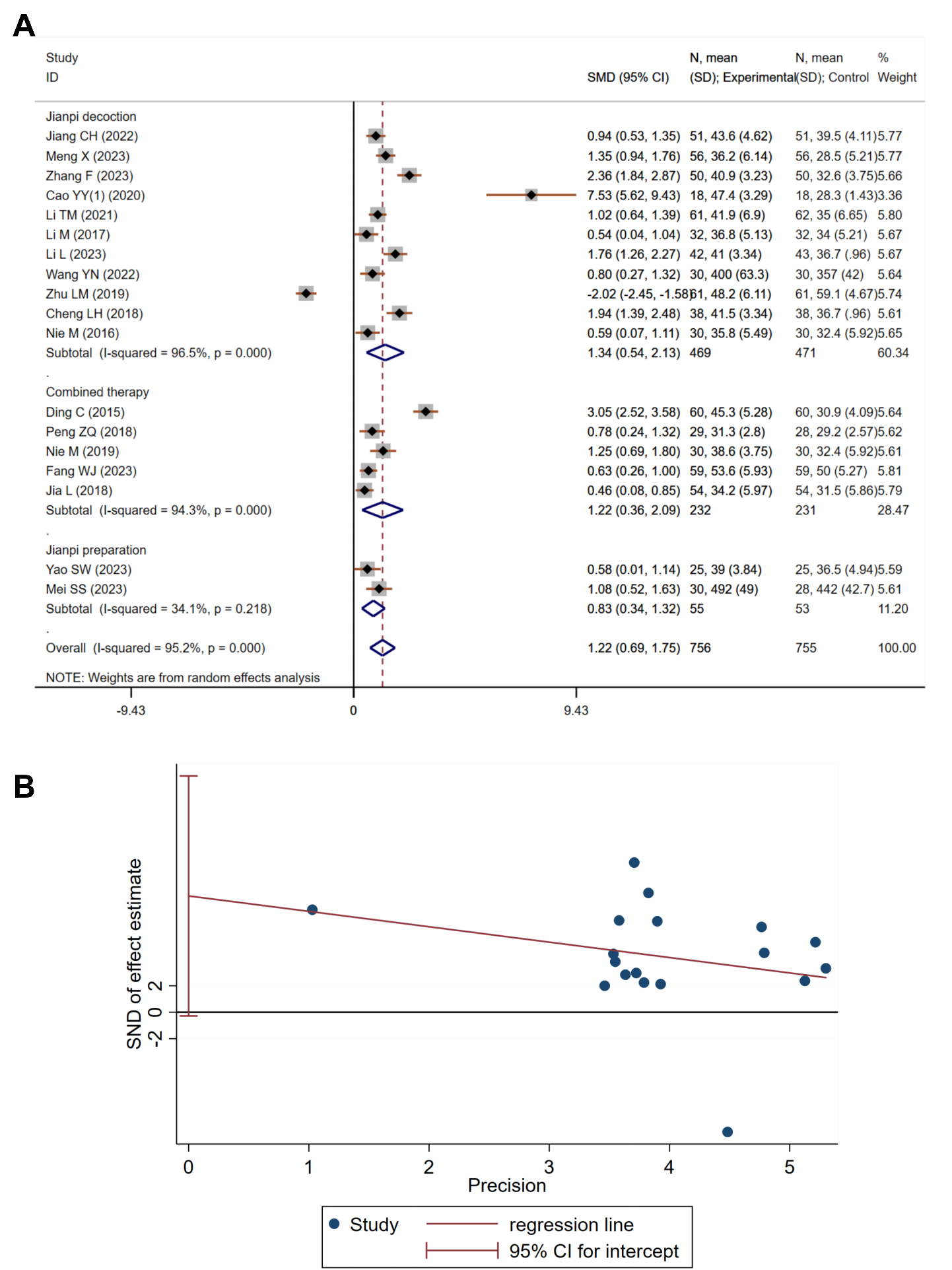
**

**Supplementary FIGURE 2. The effect of Jianpi therapy on CD4 in CRF. (A) Forrest plot. (B) Egger’s test.**

**
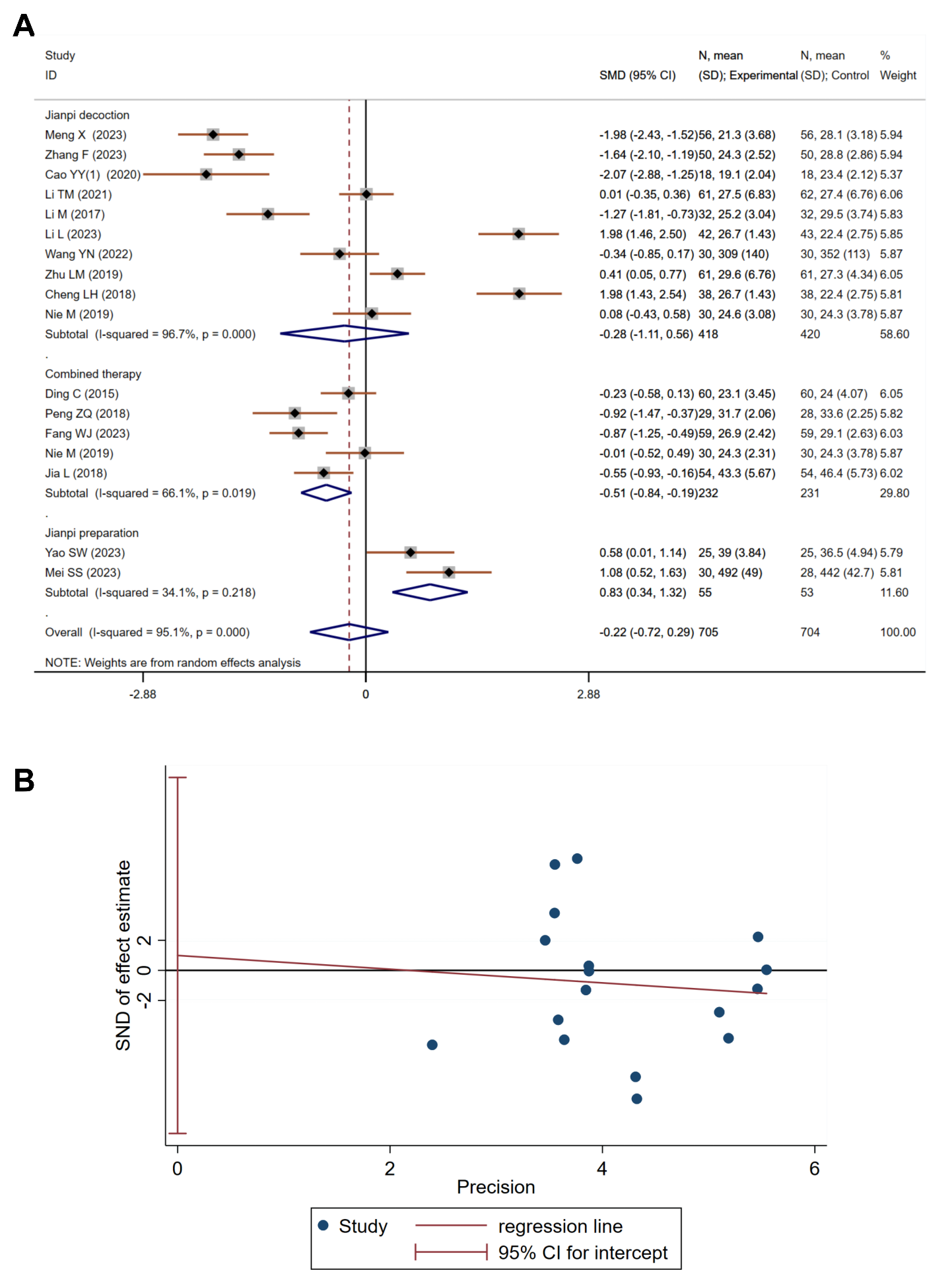
**

**Supplementary FIGURE 3. The effect of Jianpi therapy on CD8 in CRF. (A) Forrest plot. (B) Egger’s test.**


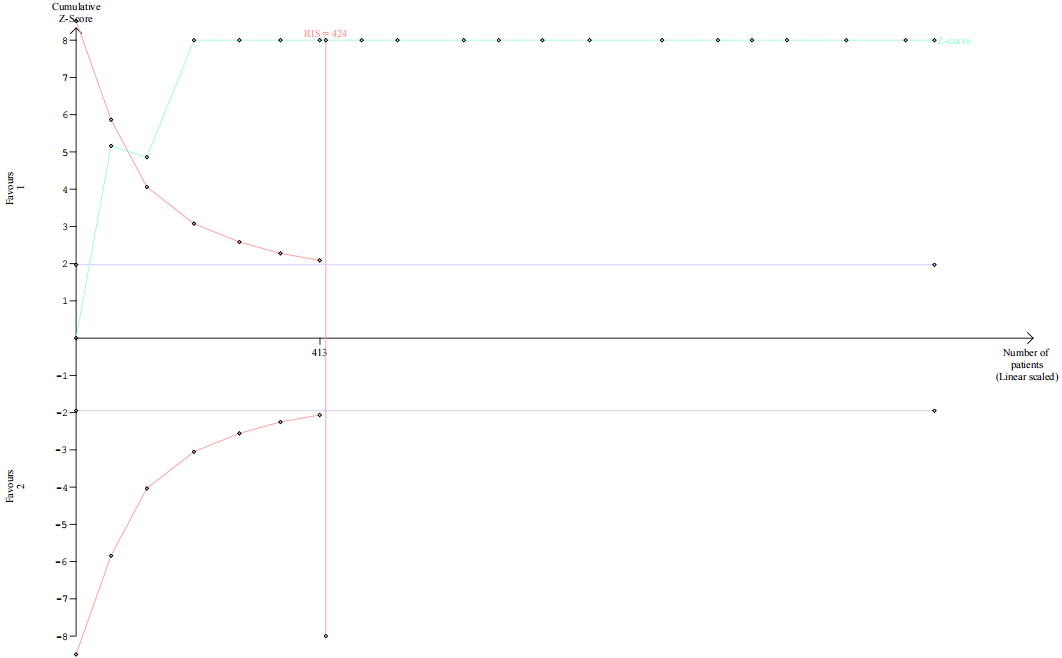


**Supplementary FIGURE 4. Test sequence analysis of PFS by Jianpi therapy.**
